# The Association Between Acylcarnitine Metabolites and Cardiovascular Disease in Chinese Patients With Type 2 Diabetes Mellitus

**DOI:** 10.3389/fendo.2020.00212

**Published:** 2020-05-05

**Authors:** Shuo Zhao, Xiao-Fei Feng, Ting Huang, Hui-Huan Luo, Jian-Xin Chen, Jia Zeng, Muyu Gu, Jing Li, Xiao-Yu Sun, Dan Sun, Xilin Yang, Zhong-Ze Fang, Yun-Feng Cao

**Affiliations:** ^1^Department of Pathology, The First Affiliated Hospital of Jinzhou Medical University, Jinzhou, China; ^2^Department of Toxicology and Sanitary Chemistry, School of Public Health, Tianjin Medical University, Tianjin, China; ^3^Shanghai Engineering Research Center of Reproductive Health Drug and Devices, NHC Key Laboratory of Contraceptives and Devices, Shanghai Institute of Planned Parenthood Research, Shanghai, China; ^4^Central Laboratory of Preventive Medicine, School of Public Health, Tianjin Medical University, Tianjin, China; ^5^Department of Epidemiology and Biostatistics, School of Public Health, Tianjin Medical University, Tianjin, China; ^6^Key Laboratory of Liaoning Tumor Clinical Metabolomics (KLLTCM), Jinzhou, China; ^7^College of Life Sciences, NanKai University, Tianjin, China; ^8^Tianjin Key Laboratory of Environment, Nutrition and Public Health, Tianjin, China

**Keywords:** type 2 diabetes mellitus, cardiovascular disease, acylcarnitine, metabolism, Chinese

## Abstract

**Objective:** The association between acylcarnitine metabolites and cardiovascular disease (CVD) in type 2 diabetes mellitus (T2DM) remains uncertain. This study aimed to investigate associations between acylcarnitines and CVD in Chinese patients with T2DM.

**Methods:** A cross-sectional study was conducted from May 2015 to August 2016. Medical records of 741 patients with T2DM were retrieved from the main electronic database of Liaoning Medical University First Affiliated Hospital. CVD was defined as having either coronary artery disease (CAD) or heart failure (HF) or stroke. Mass Spectrometry was utilized to measure levels of 25 acylcarnitine metabolites in fasting plasma. Factor analysis was used to reduce the dimensions and extracted factors of the 25 acylcarnitine metabolites. Multivariable binary logistic regression was used to obtain odds ratios (OR) of the factors extracted from the 25 acylcarnitine metabolites and their 95% confidence intervals (CI) for CVD.

**Results:** Of the 741 patients with T2DM, 288 had CVD. Five factors were extracted from the 25 acylcarnitines and they accounted for 65.9% of the total variance. Factor 1 consisted of acetylcarnitine, butyrylcarnitine, hydroxylbutyrylcarnitine, glutarylcarnitine, hexanoylcarnitine, octanoylcarnitine, and tetradecanoyldiacylcarnitine. Factor 2 consisted of decanoylcarnitine, lauroylcarnitine, myristoylcarnitine, 3-hydroxyl-tetradecanoylcarnitine, tetradecenoylcarnitine, and 3-hydroxypalmitoylcarnitine. After adjusting for potential confounders, increased factor 1 and 2 were associated with increased risks of CVD in T2DM (OR of factor 1: 1.45, 95% CI: 1.03–2.03; OR of factor 2: 1.23, 95% CI: 1.02–1.50).

**Conclusions:** Elevated plasma levels of some acylcarnitine metabolites, i.e., those extracted into factor 1 and 2, were associated with CVD risk in T2DM.

## Introduction

Cardiovascular disease (CVD) is the most common and severe diabetes complication, leading to substantial death, and disability among patients with type 2 diabetes mellitus [T2DM; (1, 2)]. Patients with T2DM are at two to three times higher risk of CVD than those without T2DM (3, 4). Compared with traditional factors such as blood pressure, lipids, and body mass index [BMI; (5, 6)], endogenous small molecule compounds detected by metabolomics approaches are more likely to reflect the cellular state related to joint effects of cell nutrition, drugs, environmental pollutants, and other external factors (7). Acylcarnitine metabolites are a group of ester substances formed by combination of free carnitine and acyl-coenzyme A (acyl-CoA) produced by fatty acids (8). A cohort study found that plasma concentrations of certain acylcarnitines were higher in T2DM patients than in non-T2DM population (9). Moreover, the concentration of acylcarnitines and mitochondrial 4-hydroxynonenal in cardiac tissue of fructose-fed mice was higher than that in the control group (10). As 4-hydroxynonenal is produced by lipid peroxidation, increased 4-hydroxynonenal may suggest high oxidative stress (11). However, only a few clinical studies investigated associations between acylcarnitines and CVD in T2DM. Indeed, it remains inconclusive whether there are associations between acylcarnitines and CVD in T2DM.

Therefore, we conducted a hospital-based cross-sectional survey to estimate the association between acylcarnitine metabolites and CVD in Chinese patients with T2DM.

## Materials and Methods

### Study Method and Population

This study used a cross-sectional study design to explore the association between acylcarnitines and CVD in T2DM. We retrieved electronic medical records of 2,554 inpatients with available metabolite data from the main electronic database of Liaoning Medical University First Affiliated Hospital (LMUFAH), Jinzhou, China who were admitted to the hospital from May 2015 to August 2016 (12).

The inclusion criteria were set out as follows: (1) age ≥ 18 years; (2) diagnosed as T2DM; and (3) acylcarnitine metabolites were available: acetylcarnitine (C2), propionylcarnitine (C3), butyrylcarnitine (C4), hydroxylbutyrylcarnitine (C4-OH), succinylcarnitine (C4DC), isovalerylcarnitine (C5), 3-hydroxyisovalerylcarnitine (C5-OH), glutarylcarnitine (C5DC), tiglylcarnitine (C5:1), hexanoylcarnitine (C6), octanoylcarnitine (C8), decanoylcarnitine (C10), lauroylcarnitine (C12), myristoylcarnitine (C14), 3-hydroxyl-tetradecanoylcarnitine (C14-OH), tetradecanoyldiacylcarnitine (C14DC), tetradecenoylcarnitine (C14:1), palmitoylcarnitine (C16), 3-hydroxypalmitoylcarnitine (C16-OH), 3-hydroxypalmitoleylcarnitine (C16:1-OH), octadecanoylcarnitine (C18), arachidic carnitine (C20), behenic carnitine (C22), tetracosanoic carnitine (C24), hexacosanoic carnitine (C26). The exclusion criteria were: (1) diagnosed as type 1 diabetes; (2) pregnancy. A total of 741 T2DM patients who met the inclusion and did not have the exclusion criteria were included in the analysis.

Ethics approval of the study was obtained from the Ethics Committee for Clinical Research of LMUFAH and informed consent was waivered by the Ethics Committee for Clinical Research of LMUFAH due to the retrospective nature of the cross-sectional study.

### Data Collection and Clinical Definition

Demographic and clinical data were retrieved from the main electronic database of the hospital, including age, gender, duration of diabetes, diabetes complications, drugs usage (antidiabetic drugs, antihypertensive drugs, and lipid lowering drugs), BMI, systolic blood pressure (SBP), diastolic blood pressure (DBP), glycated hemoglobin (HbA1c), triglyceride (TG), low-density lipoprotein cholesterol (LDL-C), and high-density lipoprotein cholesterol (HDL-C).

CVD was defined as having prior coronary artery disease (CAD), heart failure (HF), or stroke. BMI was calculated as body weight in kilograms divided by the squared body height in meters, expressed as kg/m^2^. Overweight was defined as BMI ≥ 24.0 kg/m^2^ but <28.0 kg/m^2^, and obesity defined as BMI ≥ 28.0 kg/m^2^ as recommended by the Chinese Diabetes Association for use in Chinese (13). Calibrated mercury sphygmomanometer was used to measure SBP and DBP after patients resting for 5–10 min. Overnight fasting blood (at least 8 h of fasting) was taken from T2DM patients. Measurements of HbA1c, TG, HDL-C, and LDL-C were performed in the biochemical laboratory of LMUFAH. According to the number of carbon atoms in the acyl group chain, acylcarnitines were classified into three groups: short-chain acylcarnitines including C2, C3, C4, C4-OH, C5, C5-OH, C5DC, C5:1, and C6; medium-chain acylcarnitines including C8, C10, C12, C14, C14-OH, C14DC, and C14:1; long-chain acylcarnitines including C16, C16-OH, C16:1-OH, C18, C20, C22, C24, and C26 (14).

### Acylcarnitine Quantification

The metabolomics assay method had been described in previous studies (15). Briefly, dry blood spot samples collected by finger puncture after 8 h fasting were used the metabolomics assay. The metabolomics in dry blood spots were measured using mass spectrometry (MS) technology. The MS metabolomic analysis was conducted using an AB Sciex 4000 QTrap system (AB Sciex, Framingham, MA, USA). Electrospray ionization source was ion source. The ion spray voltage was 4.5 kV. Positive mode was performed to scan analytes. The mobile phase which carried the component to be tested was 80% acetonitrile aqueous solution. Isotope-labeled internal standards of acylcarnitine from Cambridge Isotope Laboratories (Tewksbury, MA, USA) were used for absolute quantification.

### Statistical Analysis

Statistical analysis was performed using Statistical Analysis System Release 9.4 (SAS Institute Inc., Cary, NC, USA). A *P* < 0.05 was considered as statistically significant. Categorical data between two groups were compared by Chi-square test or Fisher's exact-test where appropriate and expressed as quantity and percentage. The P-P plot or Q-Q plot was used to normality tests when the analysis variable was a continuous variable. Continuous variables conformed to be the normal distribution were presented as mean ± standard deviation (SD) and compared by Student's *t*-test, or as median with interquartile range (IQR) otherwise and compared by Wilcoxon Signed Rank Test.

To cope with multiple comparisons, factor analysis was used to reduce a large number of correlated acylcarnitines to a smaller number of uncorrelated factors. Kaiser-Meyer-Olkin (KMO) and Bartlett sphericity tests were used to evaluate the suitability for factor analysis (16). A KMO coefficient < 0.5 is considered to be unacceptable while values around 0.8 were considered as meritorious. Principal component analysis was used to extract factors and to obtain the corresponding factor loading matrix. Varimax rotation rotated the initial factor load matrix to obtain a solution that is more concise and easier to interpret than the initial factor extraction (17). Individual acylcarnitines that had the maximum loading for a factor were used as relevant components of the factor. Scree plot is a line plot of the eigenvalues of factors in factor analysis (18). The horizontal axis of the scree plot is the number of factors, and the vertical axis is the eigenvalue of factors. The number of acylcarnitine factors was determined by eigenvalue, communalities, and scree plot: eigenvalue > 1, communalities ≥ 50%, number of factors located on the steep slope of the scree plot.

Multivariable binary logistic regression was used to estimate odds ratios (OR) and their 95% confidence intervals (CI) of the extracted acylcarnitine factors for CVD in T2DM. A structured adjustment scheme was applied to control for confounding effects of demographic and clinical variables. Specifically, model 1 was univariable model; model 2 was adjusted for any other acylcarnitine factors; model 3 was further adjusted for age, gender, BMI, duration of diabetes, HbA1c, SBP, DBP, TG, LDL-C, HDL-C; and model 4 further adjusted for drug usage, including antidiabetic drugs, antidiabetic drugs and lipid lowering drugs in addition to the variables adjusted in model 3.

## Result

### Description of Study Subjects

The mean age of the study patients was 57.9 (SD: 14.1) years, and median duration of diabetes was 5 (IQR: 0–10) years. Of them, 391 (52.8%) were male and 288 (38.9%) had CVD (87 with CAD alone, 109 with stroke alone, 6 with HF alone, 51 with both CAD and stroke, 53 with both CAD and HF, 18 with both stroke and HF, and 18 with all) (Table 1, Data Sheet 1).

**Table 1:** Demographic and clinical characteristics of diabetes according to occurrence of CVD.

**Variables**	**CVD**	**Non-CVD**	***P***
*N*	288 (38.9%)	453 (61.1%)	
Age, years	65.30 ± 11.05	53.19 ± 13.76	<0.0001^*^
Duration of diabetes, years	8 (2–12)	4 (0–10)	<0.0001^***^
Male gender	155 (53.8%)	236 (52.1%)	0.6471^**^
BMI, kg/m^2^	25.1 ± 3.7	25.4 ± 3.8	0.2592^*^
BMI categories			0.0738^**^
<24	102 (35.4%)	164 (36.2%)	
24~28	137 (47.6%)	184 (40.6%)	
≥28	49 (17.0%)	105 (23.2%)	
SBP, mmHg	144.86 ± 24.57	137.03 ± 22.94	<0.0001^*^
DBP, mmHg	83.13 ± 15.20	82.03 ± 12.55	0.3060^*^
HbA1c, %	8.94 ± 2.31	9.87 ± 2.32	<0.0001^*^
HbA1c categories			0.0095^**^
HbA1c ≥ 7	160 (55.6%)	315 (69.5%)	
HbA1c <7	33 (11.5%)	33 (7.3%)	
Missing value	95 (57.3%)	105 (23.2%)	
TG, mmol/L	1.62 (1.09–2.22)	1.70 (1.12–2.61)	0.0575^***^
TG categories			0.2561^**^
TG ≥ 1.7	132 (45.8%)	227 (50.1%)	
TG <1.7	156 (54.2%)	226 (49.9%)	
LDL-C, mmol/L	2.76 ± 0.90	2.97 ± 1.07	0.0037^*^
LDL-C categories			0.0364^**^
LDL-C ≥ 2.6	155 (53.8%)	279 (61.6%)	
LDL-C <2.6	133 (46.2%)	174 (38.4%)	
HDL-C, mmol/L	1.08 ± 0.36	1.09 ± 0.34	0.9037^*^
HDL-C categories			0.6315^**^
<1 in male or <1.3 in female	189 (65.6%)	305 (67.3%)	
≥1 in male or ≥1.3 in female	99 (34.4%)	148 (32.7%)	
Insulin	193 (67.0%)	372 (82.1%)	<0.0001^**^
Other antidiabetic drugs	149 (51.7%)	280 (61.8%)	0.0068^**^
Statin	161 (55.9%)	145 (32.0%)	<0.0001^**^
Other lipid lowering drugs	3 (1.0%)	15 (3.3%)	0.0505^**^
ACEIs	56 (19.4%)	53 (11.7%)	0.0037^**^
ARBs	56 (19.4%)	55 (12.1%)	0.0066^**^
Other antihypertensive drugs	154 (54.5%)	98 (21.6%)	<0.0001^**^
CAD alone	87 (30.2%)		
Stroke alone	109 (37.8)		
HF alone	6 (2.1%)		
Both CAD and stroke	51 (17.8%)		
Both CAD and HF	53 (18.4%)		
Both stroke and HF	18 (6.3%)		
CAD, stroke, and HF	18 (6.3%)		

*CVD, cardiovascular disease; BMI, body mass index; SBP/DBP, systolic/diastolic blood pressure; HbA1c, glycated hemoglobin; TG, triglyceride; LDL-C, low density lipoprotein cholesterol; HDL-C, high density lipoprotein cholesterol; ACEIs, angiotensin-converting enzyme inhibitors; ARBs, angiotensin II receptor antagonists; CAD, Coronary artery disease; HF, heart failure*.

*Data are represented as n (%), means ± standard deviation, median (interquartile range)*.

* *P-values for comparisons between groups derived by Student's t-test*.

** *P-values for comparisons between groups derived by Chi squared test*.

*** *P-values for comparisons between groups derived by Wilcoxon Signed Rank Test*.

Compared with the non-CVD group, T2DM patients with CVD were more likely to be older and had longer duration of diabetes, higher SBP, lower HbA1c, and less likely to use insulin. These patients tended to have lower LDL-C, lower rates of use of antidiabetic drugs, higher rates of use of lipid lowering drugs and antihypertensive drugs. There were no significant differences in gender, BMI, DBP, HDL-C, and TG between the two groups. C2, C4, C6, C8, C10, C12, C14, C14-OH, and C14:1 were higher while C5-OH and C24 were lower in patients with CVD than their counterparts without. Other acylcarnitines were similar in the two groups (Table 2).

**Table 2:** Acylcarnitine profile in T2DM patients.

**Variables**	**CVD**	**Non-CVD**	***P***
	**Median (interquartile range)**	**Median (interquartile range)**	
C2, μmol/L	12.620 (9.062–16.323)	11.220 (8.687–14.311)	0.0019
C3, μmol/L	1.360 (0.959–1.829)	1.350 (0.963–1.927)	0.5914
C4, μmol/L	0.216 (0.160–0.290)	0.198 (0.150–0.266)	0.0040
C4-OH, μmol/L	0.100 (0.078–0.150)	0.103 (0.076–0.150)	0.6878
C4DC, μmol/L	0.682 (0.520–0.861)	0.634 (0.464–0.835)	0.0775
C5, μmol/L	0.140 (0.110–0.193)	0.150 (0.110–0.195)	0.6370
C5-OH, μmol/L	0.250 (0.183–0.347)	0.275 (0.210–0.361)	0.0021
C5DC, μmol/L	0.080 (0.051–0.120)	0.080 (0.050–0.112)	0.2088
C5:1, μmol/L	0.060 (0.047–0.080)	0.060 (0.049–0.080)	0.7789
C6, μmol/L	0.057 (0.040–0.070)	0.046 (0.030–0.062)	<0.0001
C8, μmol/L	0.073 (0.050–0.118)	0.060 (0.040–0.090)	0.0001
C10, μmol/L	0.103 (0.070–0.160)	0.080 (0.058–0.137)	0.0001
C12, μmol/L	0.060 (0.040–0.080)	0.050 (0.040–0.070)	0.0046
C14, μmol/L	0.070 (0.051–0.100)	0.063 (0.050–0.083)	0.0067
C14-OH, μmol/L	0.054 (0.040–0.070)	0.050 (0.040–0.066)	0.0468
C14DC, μmol/L	0.040 (0.030–0.057)	0.041 (0.030–0.058)	0.4572
C14:1, μmol/L	0.100 (0.080–0.144)	0.090 (0.065–0.120)	<0.0001
C16, μmol/L	0.940 (0.700–1.210)	0.920 (0.748–1.130)	0.5651
C16-OH, μmol/L	0.030 (0.020–0.040)	0.026 (0.020–0.038)	0.2164
C16:1-OH, μmol/L	0.058 (0.046–0.073)	0.055 (0.041–0.070)	0.1642
C18, μmol/L	0.470 (0.370–0.618)	0.440 (0.360–0.570)	0.0917
C20, μmol/L	0.050 (0.038–0.060)	0.049 (0.039–0.060)	0.8092
C22, μmol/L	0.077 (0.060–0.095)	0.077 (0.060–0.098)	0.5748
C24, μmol/L	0.050 (0.040–0.070)	0.060 (0.042–0.071)	0.0107
C26, μmol/L	0.034 (0.028–0.045)	0.038 (0.030–0.050)	0.0912

*C2, acetylcarnitine; C3, propionylcarnitine; C4, butyrylcarnitine; C4-OH, hydroxylbutyrylcarnitine; C4DC, succinylcarnitine; C5, isovalerylcarnitine; C5-OH, 3-hydroxyisovalerylcarnitine; C5DC, glutarylcarnitine; C5:1, tiglylcarnitine; C6, hexanoylcarnitine; C8, octanoylcarnitine; C10, decanoylcarnitine; C12, lauroylcarnitine; C14, myristoylcarnitine; C14-OH, 3-hydroxyl-tetradecanoylcarnitine; C14DC, tetradecanoyldiacylcarnitine; C14:1, tetradecenoylcarnitine; C16, palmitoylcarnitine; C16-OH, 3-hydroxypalmitoylcarnitine; C16:1-OH, 3-hydroxypalmitoleylcarnitine; C18, octadecanoylcarnitine; C20, arachidic carnitine; C22, behenic carnitine; C24, tetracosanoic carnitine; C26, hexacosanoic carnitine*.

### Extracted Factors of Acylcarnitines

**Figure 1:**
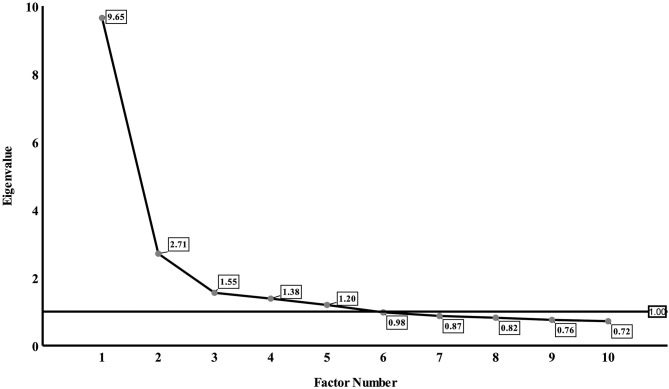
Eigenvalue of factors on scree plot. The horizontal axis of the scree plot is the number of factors, and the vertical axis is the eigenvalue of factors. Five factors had eigenvalues of more than 1.

Results of the factor analysis were acceptable as suggested by a high KMO coefficient of 0.898 and a highly significant *P*-value of Bartlett sphericity test of <0.0001. Factors 1–5 had eigenvalues of more than 1 and were located on the steep slope of the scree plot (Figure 1). So, we extracted five factors and the loadings of acylcarnitines on the five factors after varimax rotation were listed in Table 3. Factor 1 included C2, C4, C4-OH, C5DC, C6, C8, and C14DC; Factor 2 included C10, C12, C14, C14-OH, C14:1, and C16-OH; Factor 3 included C16, C16:1-OH, C18, C20, and C3; Factor 4 included C22, C24, and C26; Factor 5 included C4DC, C5, C5-OH, and C5:1. The five factors explained 65.9% of the total variance.

**Table 3:** Factor and their loadings derived by 25 acylcarnitine metabolites.

**Variables**	**Factor 1**	**Factor 2**	**Factor 3**	**Factor 4**	**Factor 5**
C2	**0.834**	0.149	0.354	0.101	0.139
C4	**0.809**	0.182	0.174	−0.002	0.341
C6	**0.773**	0.487	0.082	0.024	0.137
C5DC	**0.761**	0.408	0.064	0.147	0.031
C8	**0.692**	0.627	−0.007	0.070	0.062
C14DC	**0.665**	0.086	0.243	0.456	0.040
C4-OH	**0.642**	0.137	0.308	0.014	0.100
C12	0.231	**0.855**	0.155	0.043	0.135
C10	0.468	**0.770**	−0.031	0.100	0.054
C14:1	0.538	**0.654**	0.118	0.126	0.072
C14	0.131	**0.652**	0.476	−0.068	0.215
C14-OH	0.227	**0.532**	0.228	0.068	0.095
C16-OH	−0.085	**0.394**	0.384	0.117	0.158
C18	0.224	0.165	**0.800**	0.164	0.135
C16	0.261	0.217	**0.763**	0.045	0.159
C16:1-OH	0.170	0.149	**0.676**	0.283	0.089
C20	0.152	0.059	**0.476**	0.309	0.042
C3	0.198	−0.003	**0.474**	0.174	0.459
C24	−0.001	0.180	0.150	**0.800**	0.133
C26	0.070	0.021	0.153	**0.778**	0.145
C22	0.136	−0.039	0.351	**0.684**	0.005
C5-OH	−0.040	0.193	0.218	0.090	**0.735**
C5	0.439	0.079	0.191	0.088	**0.630**
C4DC	0.111	0.088	−0.116	0.429	**0.510**
C5:1	0.400	0.180	0.185	0.027	**0.492**

*C2, acetylcarnitine; C3, propionylcarnitine; C4, butyrylcarnitine; C4-OH, hydroxylbutyrylcarnitine; C4DC, succinylcarnitine; C5, isovalerylcarnitine; C5-OH, 3-hydroxyisovalerylcarnitine; C5DC, glutarylcarnitine; C5:1, tiglylcarnitine; C6, hexanoylcarnitine; C8, octanoylcarnitine; C10, decanoylcarnitine; C12, lauroylcarnitine; C14, myristoylcarnitine; C14-OH, 3-hydroxyl-tetradecanoylcarnitine; C14DC, tetradecanoyldiacylcarnitine; C14:1, tetradecenoylcarnitine; C16, palmitoylcarnitine; C16-OH, 3-hydroxypalmitoylcarnitine; C16:1-OH, 3-hydroxypalmitoleylcarnitine; C18, octadecanoylcarnitine; C20, arachidic carnitine; C22, behenic carnitine; C24, tetracosanoic carnitine; C26, hexacosanoic carnitine*.

*The factors were extracted from 25 acylcarnitine according to eigenvalue > 1, scree plot and cumulative variance by principal component analysis. Varimax rotation was used to maximize the variance difference of each factor, so as to better explain the factors. Each black-marked acylcarnitine in each column is classified as a factor in the corresponding column*.

### Associations Between Extracted Factors and CVD Risk in T2DM

Factors 1, 2, and 4 were all positively associated with CVD risk in T2DM in univariate analysis. After adjustment for other factors, these positive associations were still significant (Model 2). However, only factor 1 (OR: 1.42, 95% CI: 1.03–1.95) and factor 2 (OR: 1.24, 95% CI: 1.03–1.49) were still positively associated with risk of CVD after further adjustment for age, sex, BMI, duration of diabetes, HbA1c, SBP, DBP, TG, LDL-C, and HDL-C. After the final adjustment for usage of drugs, the effect size of factor 1 (OR: 1.45, 95% CI: 1.03–2.03) and factor 2 (OR: 1.23, 95% CI: 1.02–1.50) were largely unchanged (Table 4).

**Table 4:** Univariable and multivariable association of metabolomic factors with cardiovascular event.

**Model**	**Factor**	**OR**	**95%CI**	***P***
Model 1	Factor 1	1.44	1.12–1.86	0.0047
	Factor 2	1.25	1.08–1.45	0.0034
	Factor 3	1.10	0.94–1.26	0.2461
	Factor 4	0.85	0.73–0.99	0.0354
	Factor 5	0.87	0.75–1.01	0.0604
Model 2	Factor 1	1.75	1.31–2.34	0.0002
	Factor 2	1.38	1.16–1.64	0.0003
	Factor 3	1.12	0.96–1.31	0.1397
	Factor 4	0.82	0.70–0.96	0.0155
	Factor 5	0.88	0.75–1.02	0.0951
Model 3	Factor 1	1.42	1.03–1.95	0.0322
	Factor 2	1.24	1.03–1.49	0.0238
	Factor 3	1.12	0.94–1.34	0.2023
	Factor 4	0.89	0.74–1.06	0.1910
	Factor 5	0.96	0.80–1.16	0.6833
Model 4	Factor 1	1.45	1.03–2.03	0.0330
	Factor 2	1.23	1.02–1.50	0.0344
	Factor 3	1.17	0.97–1.41	0.1069
	Factor 4	0.86	0.71–1.04	0.1201
	Factor 5	0.97	0.80–1.17	0.7242

*Model 1: Univariable model; Model 2: Multivariable model, adjusted for other acylcarnitine factors; Model 3: Multivariable model, further adjusted for age, sex, body mass index, Duration of diabetes, glycated hemoglobin, systolic blood pressure, diastolic blood pressure, triglyceride, low density lipoprotein cholesterol, and high density lipoprotein cholesterol; Model 4: Multivariable model, further adjusted for antidiabetic drugs, lipid lowering drugs, and antihypertensive drugs*.

### Sensitivity Analysis

After mean imputation and multiple imputation of missing values in HbA1c (*n* = 200), the effect sizes of factors 1 and 2 for CVD risk in T2DM remained stable and significant in uni- and multi-variable analyses (Table S1).

## Discussion

This study showed that some acylcarnitines, i.e., C2, C4, C4-OH, C5DC, C6, C8, and C14DC in factor 1, and C10, C12, C14:1, C14, C14-OH, and C16-OH in factor 2, were associated with the risk of CVD in T2DM and the associations were independent of other acylcarnitines factors and traditional CVD risk factors. These acylcarnitine metabolites extracted in factor 1 and 2 were mainly short and medium-chain acyl-carnitines.

**Figure 2:**
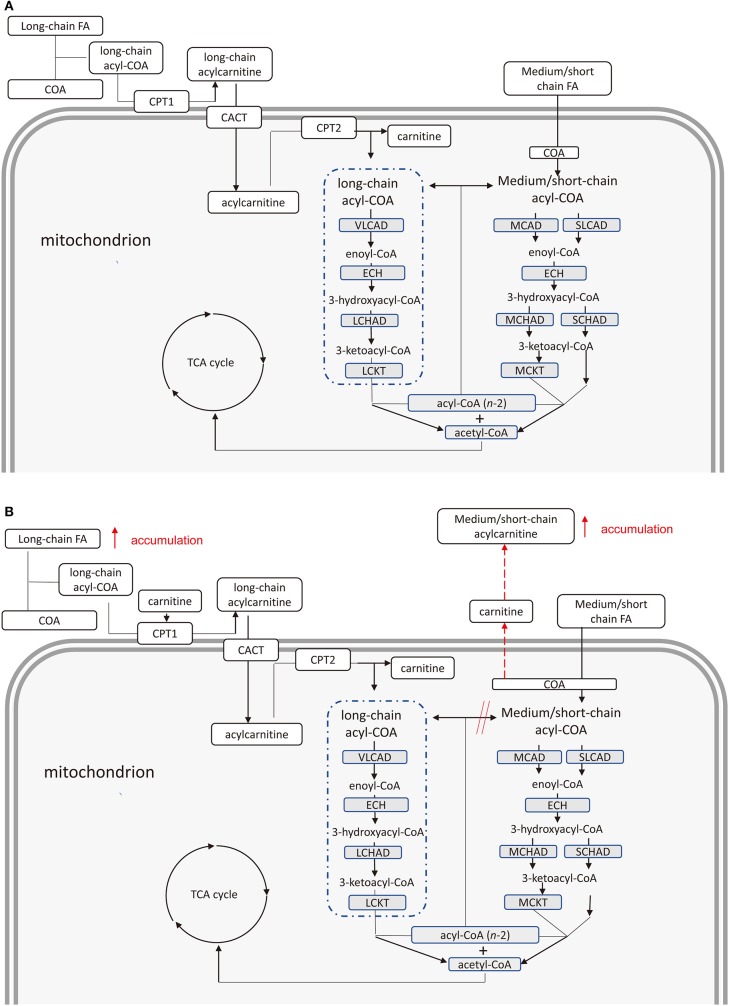
**(A)** Pathway of fatty acids provide energy in health cardiomyocytes. Chain fatty acids provide energy through the production of acetyl coenzyme A under the combined catalysis of several enzymes. **(B)** Pathway of fatty acids provide energy in cardiomyocytes of type 2 diabetes mellitus patients with cardiovascular disease. Accumulation of free long-chain fatty acids inhibits complete oxidation of medium and short-chain fatty acids leading to increased medium and short-chain acyl-COA. Medium and short-chain acylcarnitine increases due to the combination of these acyl-COA and carnitine. FA, fatty acid; COA, Coenzyme A; CPT 1, carnitine palmitoyltransferase 1; CACT, carnitine acylcarnitine translocase; CPT 2, carnitine palmitoyltransferase 2; VLCAD, very long-chain acyl-coA dehydrogenase; ECH, enoyl-CoA hydratase; LCHAD, long-chain 3-hydroxyacyl-CoA-dehydrogenase; LCKAT, long-chain ketoacyl-CoA Thiolase; MCAD, medium-chain acyl-CoA dehydrogenase; MCKAT, medium-chain 3-ketoacyl-CoA thiolase; TCA, tricarboxylic acid.

The energy supply pathway of long chain fatty acid oxidation in human is as follows. In cells, long-chain fatty acid (FA) combines with coenzyme A (COA) to form long-chain acyl-coenzyme A (acyl-COA) (19). Carnitine combines with acyl group of long-chain acyl-COA to form long-chain acylcarnitine by carnitine palmitoyltransferase 1 located in outer mitochondrial membrane (20). Acylcarnitine are transported into mitochondria via carnitine acylcarnitine translocase (CATC) (21). Long-chain acylcarnitine is decomposed into long-chain acyl-CoA and carnitine by carnitine palmitoyltransferase 2 in inner mitochondrial membrane (22). Long-chain acyl-COA is dehydrogenated under the action of very long-chain acyl-COA dehydrogenase to form enoyl-COA (23). Enoyl-COA produces 3-hydroxyacyl-CoA through hydration under regulation of enoyl-CoA hydratase (23). 3-hydroxyacyl-CoA is dehydrogenated by long-chain 3-Hydroxyacyl-CoA-Dehydrogenase to form 3-ketoacyl-CoA (24). The 3-ketoacyl-CoA undergoes thiolysis by long-chain letoacyl-CoA thiolase to produce an acetyl-CoA and a two-carbon chain shortened acyl-CoA (25). The shortened COA re-enters this metabolic cycle until which enters the oxidation pathway of short-chain fatty acids. Compared with long-chain fatty acids, medium and short-chain fatty acid can enter mitochondria directly without the assistance of CACT. Similar to long-chain fatty acids, medium and short-chain fatty acids produce shortened-COA compared and acetyl-CoA under the combined catalysis of some similar enzymes. The shortened-COA re-enters the short-chain fatty acid cycle until it is converted to acetyl-CoA. Finally, acetyl-CoA produced in oxidation of fatty acid provides energy by participating in tricarboxylic acid cycle (Figure 2A).

Several studies reported decreased rates of glucose oxidation, increased oxidation rates of FA and myocardial oxygen consumption in ob/ob and db/db mice (26, 27). An American research team drew a similar conclusion by repeatedly measuring above mice at different week of age (28). The team suspected, through further PCR experiment, that gene expression involved in FA uptake and oxidation were out of balance, which led to FA entered cardiomyocytes exceeding mitochondrial oxidative capacity. In addition, some other studies found that cardiomyocytes of Zucker rats were not significantly increased in oxidation compared to increased long-chain FA in cardiomyocytes (29, 30). A study determined human FA levels and found that although the FA oxidation rate increased, percentage of fatty acid oxidation amount was lower due to increased intake (31). Besides, acyl-CoA can accumulate in the cytoplasm if FA overload is imposed (32). Consistently, metabolomics analysis found that compared with non-T2DM patients, oxidation of long chain fatty acids in T2DM patients were incomplete, leading to significance increased plasma levels of C6, C8, C10, C12, and C14 (33). Some researchers showed that body fat percentage correlated positively with serum levels of C2, C3, C4, C5, C6, C8:1, and C16:1 (34). It was likely that higher body fat correlated with an incomplete beta oxidation of fatty acids, which predominantly leaded to higher amounts of short or medium-chain acylcarnitine. They also found that serum levels of C2, C6, C8, C10, C12, C14, C14:1, and C14-OH were increased in prediabetic state (34).

Therefore, we suspect that a slightly elevated fatty acid oxidation rate of cardiomyocytes in patients with T2DM cannot adequately oxidize long-chain free FAs, accumulated in cardiomyocytes. The accumulated free FAs induce cardiotoxicity. On the other hand, the oxidation pathway of medium and short-chain fatty acids may be inhibited due to the increased oxidation rate of long-chain fatty acids. Due to this inhibition, the medium and short-chain coenzyme A is not sufficiently converted to acetyl-CoA. Finally, cumulative acyl-CoA binding carnitine leads to an increase in medium and short-chain acylcarnitines (Figure 2B).

There were several limitations noticed in our study. First, our study was a cross-sectional study and could not establish causal relationships between the acylcarnitines and CVD in T2DM. Second, our subjects were inpatients. Their T2DM were more severe than the patients with T2DM at large. Thus, our findings cannot be extrapolated to the general population of patients with T2DM. Third, our study did not collect dietary factors and diet habit may be one of major confounders in our analysis (35). However, we had carefully adjusted for potentially confounding effects of other acylcarnitine metabolites, and demographic and clinical factors including but not limited to age, BMI, BP, HbA1c, and lipid profile. As “outcomes” of diet, adjustment for those acylcarnitine metabolites and clinical factors may have partially removed the confounding effects of dietary habit. Nevertheless, we acknowledged that despite carful adjustment for these confounding factors, we cannot exclude that there were unadjusted confounding effects. Therefore, our results need to be interpreted with caution. Fourth, we didn't determine levels of insulin resistance and acyl-CoA. At last, 200 cases of HbA1c were missing in our subjects. However, these associations remained stable after mean imputation and multiple imputations, suggesting that a major bias is unlikely.

Our research had important public health implications. CVD is a common and serious complication in T2DM, which is significantly associated with premature death in T2DM. Although intensive management of hyperglycemia, hypertension and abnormal lipids are able to reduce the risk of CVD in T2DM, the residual risk of CVD remains substantially high (36). It is important to better understand mechanisms of CVD in T2DM. Our study provides a new insight about pathways from metabolic disturbances in T2DM to CVD.

In conclusion, our study found that elevated plasma levels of some acylcarnitine metabolites extracted in factors 1 and 2, i.e., C2, C4, C6, C8, C10, C12, C14, C14OH, and C14:1, were associated with risk of CVD in Chinese inpatients with T2DM. As the nature of the cross-sectional study, prospective cohort studies are needed to replicate our findings in the future.

## Data Availability Statement

The datasets generated for this study can be found in Metabolights, with the unique identifier MTBLS1427, accessible via http://www.ebi.ac.uk/metabolights/MTBLS1427.

## Ethics Statement

The studies involving human participants were reviewed and approved by Ethics Committee for Clinical Research of Liaoning Medical University First Affiliated Hospital. Written informed consent for participation was not required for this study in accordance with the national legislation and the institutional requirements.

## Author Contributions

Z-ZF, SZ, and XY designed the study. SZ and X-FF analyzed the data and wrote the draft. H-HL and MG collected the data. JL, Y-FC, and X-YS gave critical comments and contributed to the writing of this manuscript. SZ, TH, J-XC, JZ, and DS participated in revision of this manuscript.

## Conflict of Interest

The authors declare that the research was conducted in the absence of any commercial or financial relationships that could be construed as a potential conflict of interest.
