# Supplementary material for: The Association Between Acylcarnitine Metabolites and Cardiovascular Disease in Chinese Patients With Type 2 Diabetes Mellitus
Source: Front Endocrinol (Lausanne). 2020 May 5;11:212. doi: 10.3389/fendo.2020.00212 (PMC7214635; doi:10.3389/fendo.2020.00212)
Supplement: Supplementary file 2 [file Table_1.DOCX]

**Supplementary Table**

Table S1. Association of metabolomic factors with cardiovascular event after imputation and multiple imputation to missing value of glycated hemoglobin.

| Model |  | Mean imputation | | | Multiple imputation | | | |
| --- | --- | --- | --- | --- | --- | --- | --- | --- |
|  | Factor | OR | 95% CI | P | OR | 95% CI | | P |
| Model1 | Factor1 | 1.44 | 1.12-1.86 | 0.0047 | 1.44 | 1.12-1.86 | | 0.0047 |
|  | Factor2 | 1.25 | 1.08-1.45 | 0.0034 | 1.25 | 1.08-1.45 | | 0.0034 |
|  | Factor3 | 1.10 | 0.94-1.26 | 0.2461 | 1.10 | 0.94-1.26 | | 0.2461 |
|  | Factor4 | 0.85 | 0.73-0.99 | 0.0354 | 0.85 | 0.73-0.99 | | 0.0354 |
|  | Factor5 | 0.87 | 0.75-1.01 | 0.0604 | 0.87 | 0.75-1.01 | | 0.0604 |
| Model2 | Factor1 | 1.75 | 1.31-2.34 | 0.0002 | 1.75 | 1.31-2.34 | | 0.0002 |
|  | Factor2 | 1.38 | 1.16-1.64 | 0.0003 | 1.38 | 1.16-1.64 | | 0.0003 |
|  | Factor3 | 1.12 | 0.96-1.31 | 0.1397 | 1.12 | 0.96-1.31 | | 0.1397 |
|  | Factor4 | 0.82 | 0.70-0.96 | 0.0155 | 0.82 | 0.70-0.96 | | 0.0155 |
|  | Factor5 | 0.88 | 0.75-1.02 | 0.0951 | 0.88 | 0.75-1.02 | | 0.0951 |
| Model3 | Factor1 | 1.47 | 1.07-2.02 | 0.0175 | 1.48 | | 1.08-2.20 | 0.0153 |
|  | Factor2 | 1.23 | 1.02-1.47 | 0.0300 | 1.22 | | 1.03-1.48 | 0.0246 |
|  | Factor3 | 1.15 | 0.97-1.38 | 0.1176 | 1.14 | | 0.95-1.36 | 0.1598 |
|  | Factor4 | 0.89 | 0.74-1.07 | 0.2131 | 0.89 | | 0.74-1.07 | 0.2039 |
|  | Factor5 | 0.99 | 0.82-1.18 | 0.8776 | 0.99 | | 0.83-1.19 | 0.9537 |
| Model4 | Factor1 | 1.52 | 1.08-2.13 | 0.0164 | 1.52 | | 1.08-2.13 | 0.0154 |
|  | Factor2 | 1.22 | 1.01-1.48 | 0.0430 | 1.23 | | 1.01-1.49 | 0.0367 |
|  | Factor3 | 1.21 | 0.99-1.46 | 0.0558 | 1.19 | | 0.98-1.44 | 0.0777 |
|  | Factor4 | 0.86 | 0.71-1.04 | 0.1262 | 0.86 | | 0.71-1.04 | 0.1240 |
|  | Factor5 | 0.99 | 0.82-1.20 | 0.9241 | 0.99 | | 0.82-1.21 | 0.9796 |

Model 1: Univariable model;

Model 2: Multivariable model, adjusted for other acylcarnitine factors;

Model 3: Multivariable model, further adjusted for age, sex, body mass index, Duration of diabetes, glycated hemoglobin after imputation, systolic blood pressure, diastolic blood pressure, triglyceride, low density lipoprotein cholesterol, high density lipoprotein cholesterol;

Model 4: Multivariable model, further adjusted for antidiabetic drugs, lipid lowering drugs, antihypertensive drugs.
